# Vacuum physiotherapy after first stage buccal mucosa graft (BMG) urethroplasty in children with proximal hypospadias

**DOI:** 10.1590/S1677-5538.IBJU.2019.0845

**Published:** 2020-09-02

**Authors:** Marco Bandini, Sasha Sekulovic, Bogdan Spiridonescu, Anuj Deep Dangi, Pramod Krishnappa, Alberto Briganti, Andrea Salonia, Francesco Montorsi, Rados Djinovic

**Affiliations:** 1 Sava Perovic Foundation Center for Genito-Urinary Reconstructive Surgery Belgrade Serbia Sava Perovic Foundation, Center for Genito-Urinary Reconstructive Surgery, Belgrade, Serbia;; 2 Division of Oncology/Unit of Urology IRCCS Ospedale San Raffaele Vita-Salute San Raffaele University Milan Italy Division of Oncology/Unit of Urology, URI, IRCCS Ospedale San Raffaele, Vita-Salute San Raffaele University, Milan, Italy;; 3 FundeniClinical Institute-Center for Uronephrology and Renal Transplantation Bucharest Romania FundeniClinical Institute-Center for Uronephrology and Renal Transplantation, Bucharest, Romania;; 4 Department of Urology Christian Medical College and Hospital VelloreTamil Nadu India Department of Urology, Christian Medical College and Hospital, Vellore, Tamil Nadu, India;; 5 Department of Urology NU Hospitals Bangalore India Department of Urology, NU Hospitals, Bangalore, India

**Keywords:** Hypospadias, Mouth Mucosa, Vacuum

## Abstract

**Introduction:**

To assess the feasibility of vacuum physiotherapy meant to decrease graft contraction and recurrent penile curvature (PC), hence successful tubularization and a straight penis in patients underwent two-stage buccal mucosa graft (BMG) urethroplasty, in proximal hypospadias repair.

**Material and methods:**

Between January 2014 and July 2018, 59 two-stage BMG urethroplasties performed at our referral center, were included in the study. The parents were counseled to use the vacuum device between the two stages. An internal, self-administered, semiquantitative, non-validated questionnaire was designed to record parent and patient adherence to the vacuum physiotherapy and parent satisfaction. Success rate of graft tubularization, curvature correction rates, and status of early (4 months) postoperative urinary stream were evaluated.

**Results:**

Of 45/59 (76.3%) who returned the questionnaire, 77.8% followed the recommended physiotherapy protocol using the vacuum device. 93.3% of parents replied that the use of the vacuum was easy or moderately easy. None of the parents interrupted the physiotherapy because of perceived difficulty or intolerability. 100% of parents would have repeated the physiotherapy, if they had to. Overall, success rate of tubularization was 98.3% (58/59), complete curvature correction was achieved in 88.2% (52/59) of patients, and 79.7% (47/59) of patients showed a straight and powerful early post-operative urinary stream.

**Conclusions:**

Physiotherapy with the vacuum device is safe, easy and practically feasible. Our vacuum physiotherapy protocol had high compliance rate. Vacuum physiotherapy should be considered for further assessment in patients undergoing two stage hypospadias repair using buccal mucosa.

## INTRODUCTION

Proximal hypospadias repair represents a challenging endeavor for pediatric urologists and plastic surgeons worldwide ( 1 ). In these complex patients, single stage urethral closure is hampered by severe penile curvature (PC), scarred urethral plate and deficiency of genital tissue ( 2 ). So, two stage buccal mucosa graft (BMG) urethroplasty becomes a good viable option in such patients, where the single stage procedure is likely to result in suboptimal outcomes ( 3 ).

Nevertheless, approximately 8-10% of BMGs develop fibrosis or contracture after the first stage requiring re-grafting and one third of the graft have some degree of fibrosis/induration, which results in suboptimal outcomes after second stage urethroplasty ( 4 ). Despite the fact that BMG has least tendency to contract on exposure to urine ( 5 ), early graft contraction after first stage following good graft uptake is a well-recognized phenomenon ( 6 ). Furthermore, proximal hypospadias is associated with severe ventral PC which requires penile lengthening procedures (ventral tunica attenuations). However, ventral incisions are prone to scar formation leading to recurrent PC and consequent graft fibrosis after the first stage. In spite of these possible complications, no special precautions are usually taken after the first stage of surgery to increase the success rate of tubularization ( 6 ) and decrease the rate of recurrent PC at second stage.

We hypothesized that the vacuum induced penile erections and maintaining the moist environment, would prevent the graft contracture and maintain the pliant nature of buccal mucosa after the first stage. We also hypothesized that the vacuum would modulate the healing process after lengthening incisions, resulting in lower rate of recurrent PC after first stage, hence achieving a straight penis after second stage. In this study we aim to assess the compliance, acceptability and possible complications of vacuum therapy in our patients and report their surgical outcomes.

## MATERIALS AND METHODS

### Study population

**Figure 1:**
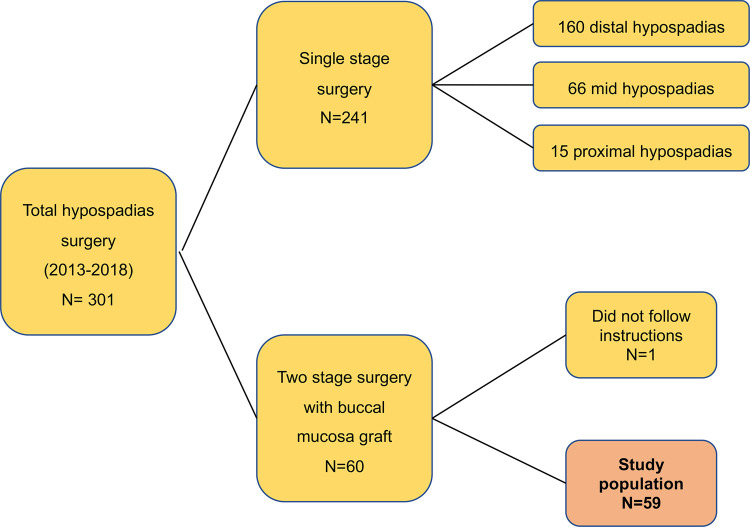
Study flow showing the selection of patient cohort.

We performed a retrospective descriptive analysis of prospectively collected data from a cohort of 60 patients, who underwent BMG urethroplasty between January 2013 and July 2018, at our center. Inclusion and exclusion criteria are specified in Figure-1 . The study was approved by our internal review board.

### Two-stage BMG urethroplasty

The two-stage BMG urethroplasty followed principles reported previously by Bracka et al. ( 7 , 8 ). Briefly, in the first stage the short urethra that caused severe ventral curvature was excised/dissected proximally to lengthen and to straighten the penis. After, PC was evaluated using artificial erection and subsequently corrected by ventral tunica attenuations, by giving superficial transverse incisions on the tunica albuginea at the site of maximum concavity of the cavernosum bodies. We harvested BMG from the inner cheek using a standard, previously described technique ( 9 ). Redundant vascularized dartos tissue was then transposed to the ventral side of the penis, over the tunica albuginea. The graft was placed ventrally from the native urethral meatus to the tip of the corporal bodies, quilting the BMG with the dartos with absorbable stitches. In the end, paraffin gauze was stitched over the BMG in order to achieve a good attachment between the graft and the vascularized dartos. Coban® dressing was placed around the penis to prevent lymphedema and the swelling of the penile skin, and it was exchanged on daily basis. The paraffin gauze was left in place for five days and Foley catheter for 7 days. Six months after the initial procedure, the child was brought back to the operating room for the second stage. Once the patient was anesthetized, the graft was carefully inspected for areas of fibrosis and induration. If the graft was deemed suitable, the margins of the BMG were marked and a U-shaped incision was made, otherwise re-grafting with or without tubularization was done. Persistent PC was assessed with artificial erection and eventually corrected by dorsal plication of the tunica albuginea after lifting the neurovascular bundle ( 10 ). Pubic lipectomy ( 11 ) was performed in patients with pubic fat hypertrophy. Subsequently, the edges of the graft were dissected and raised from the bed enough to allow tension-free tubularization. The glans was incised along the midline and both glans wings were dissected to enable glans closure without tension. The neo-meatus was created first by joining the edges of the glans wings. The rest of the graft was then tubularized. Whenever available, a local dartos flap was transposed to cover the neourethra. Urethral stent and suprapubic catheter were left in place for 14 days.

### Vacuum physiotherapy

Seven days after the first stage, the bladder catheter was removed and vacuum physiotherapy was started. Two 50cc syringes were joined with a piece of a latex catheter (made after cutting the two ends of 20 Fr Foley catheter) to create the vacuum device (Supplementary Video-1). The device was made by a member of the team, and the parents were taught how to reproduce it. One syringe was used as a cylinder and the other as a vacuum pump. We recommended 15-20vacuum induced erections of the penis twice a day followed by a massage of the BMG with Fusidic Acid cream 2% (replaced with Bepanthen® ointment two weeks after surgery) every day till second stage surgery. We created a negative suction of pulling syringe plunger up to 30cc mark, or till the penis was fully erected. Ointments were used for moisturizing the BMG, while the massage was recommended for maintaining graft elasticity. Parents were instructed on how to use the vacuum device and were asked to continue it until the second stage surgery ( 12 ).

### Study outcomes

**Figure 2:**
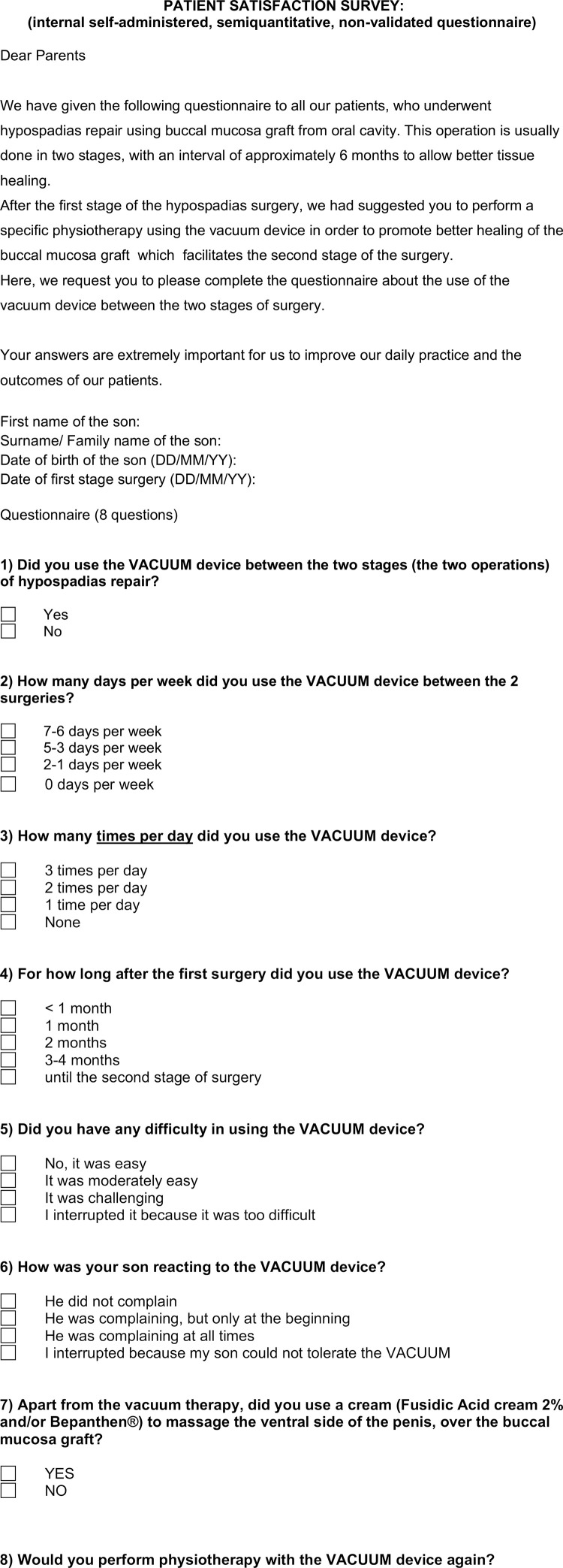
Patient survey on the use of vacuum device between the two stages of surgery that was sent to all parents.

**Figure 3:**
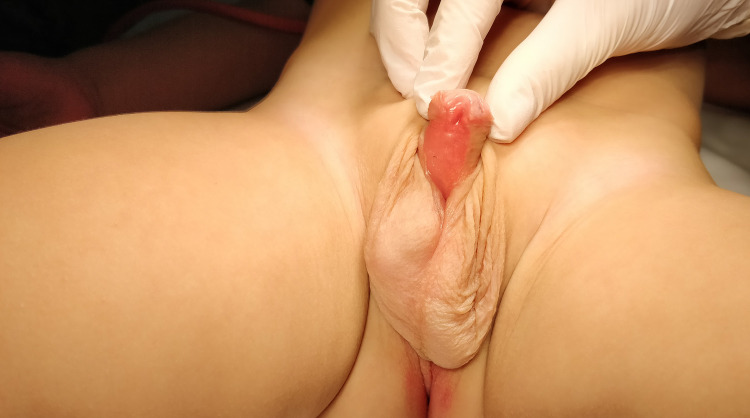
In this figure it is appreciable the ventral side of a hypospadic penis before the second stage of surgery. The BMG is smooth and trophic without any scar.

Five predefined outcomes were examined in the current study: 1) Parent and patient adherence to the vacuum physiotherapy, 2) Parent satisfaction, 3) Status of the BMG after the vacuum physiotherapy at the time of second stage surgery, 4) Proportion of patients with straight penis after the second stage, and 5) Urinary stream assessment four months after second stage BMG urethroplasty. First and second outcomes were recorded using an internal, self-administered, semiquantitative, non-validated questionnaire ( 13 ) ( Figure-2 ). Whereas, third, fourth and fifth outcomes were calculated using our surgical database. To evaluate parent and patient adherence to the vacuum physiotherapy, the questionnaire included 5 questions designed to investigate how parents used the vacuum device and 2 questions designed to investigate whether the use of the device was challenging for them or for their children. To evaluate parent satisfaction, we asked whether parents would have performed the vacuum therapy again, if they had to. Questionnaires were provided in English, Serbian, Russian and Romanian based on the spoken language of the parents. To evaluate the status of the BMG after 6 months of vacuum physiotherapy, all patients planned for the second stage of surgery were examined by two members of our surgical team. Here, grafts were classified as smooth and trophic or ‘favorable for tubularization’, when no scars or shrinkage were identified ( Figure-3 ) and ‘unfavorable for tubularization’, when such aspects were found. To evaluate the rate of straight penis after second stage, we asked parents to send postoperative photos of the penis from two angles in erection every 6 months, via email. Here, residual or recurrent PC was defined as any clinically relevant curvature, which may cause discomfort for the parents of the patients or exceed 10 degrees of bending. Moreover, to better evaluate the effect of vacuum physiotherapy after ventral tunica attenuations, we also examined the presence and the severity of recurrent PC during the second stage surgery. Finally, to evaluate early (4 months) post-operative urinary stream, we asked the parents to send a video-report of the urinary stream of their children taken in two views perpendicular to each other. All videos were examined by two members of our team (SS or MB) and the urinary streams were classified as powerful or weak, as well as straight or deflected.

## RESULTS

### Patient characteristics

Between January 2013 and July 2018, we retrospectively identified 59 hypospadias patients from our database, that were eligible for the study purpose, see Figure-1 and Table-1 . Out of 59 patients, 45 returned the filled study questionnaire. The baseline characteristics, surgical interventions and outcomes in terms of surgical complications, of those who returned the filled study questionnaire and those who did not were comparable ( Table-1 ).

**Table 1:** General patient characteristics of 59 hypospadias patients treated with BMG urethroplasty at our referral center, between January 2013 and July 2018.

Variables		Overall	Patients who did not fulfil the questionnaire (14)	Patients who fulfilled the questionnaire (45)	P values
Age at 1st stage of surgery (months)	Median	20	27	20	0.2
	Range	14-36	18.2-76	14-31	
Age at 2nd stage of surgery (months)	Median	27	34	26	0.1
	Range	21-43	25.2-83	21-39	
Time between the two stages (months)	Median	6	6	6	0.5
	Range	6-8	6-7.8	7-8	
Lipectomy	No	11 (18.6)	2 (14.3)	9 (20)	0.9
	Yes	48 (81.4)	12 (85.7)	36 (80)	
Location of the meatus	Proximal	51 (86.4)	12 (85.7)	39 (86.7)	1
	Proximal-shaft	8 (13.6)	2 (14.3)	6 (13.3)	
Previously operated for hypospadias	No	46 (78)	10 (71.4)	36 (80)	0.8
	Yes	13 (22)	4 (28.6)	9 (20)	
Penile curvature at presentation	None or < 30	1 (1.7)	0 (0)	1 (2.2)	0.6
	>60	50 (84.7)	13 (92.9)	37 (82.2)	
	30-60	8 (13.6)	1 (7.1)	7 (15.6)	
Penile curvature at second stage of surgery	None or < 30	17 (28.8)	3 (21.4)	14 (31.1)	0.4
	>60	4 (6.8)	2 (14.3)	2 (4.4)	
	30-60	38 (64.4)	9 (64.3)	29 (64.4)	
DSD	No	48 (81.4)	10 (71.4)	38 (84.4)	0.5
	Yes	11 (18.6)	4 (28.6)	7 (15.6)	
Curvature management at 1° stage	Plication	2 (3.4)	0 (0)	2 (4.4)	1
	Chordee release or ventral tunica attenuations	57 (96.6)	14 (100)	43 (95.6)	
Curvature management at 2° stage	Plication	47 (79.7)	12 (85.7)	35 (77.8)	0.8
	None	12 (20.3)	2 (14.3)	10 (22.2)	
Skin reconstruction	Direct ventral closure	46 (78)	12 (85.7)	34 (75.6)	0.7
	flaps or grafts from skin, dartos or preputial	13 (22)	2 (14.3)	11 (24.4)	
Fistula	No	32 (54.2)	11 (78.6)	21 (46.7)	0.07
	Yes	27 (45.8)	3 (21.4)	24 (53.3)	
Any complications	No	28 (47.5)	9 (64.3)	19 (42.2)	0.3

DSD = Disorder of sexual differentiation

### Adherence to the vacuum physiotherapy and parent satisfaction

Overall, 45 (76.3%) patients returned the questionnaire, hence were eligible for assessment. Questionnaire driven parent and patient adherence to the vacuum physiotherapy and parent satisfaction rates are summarized in Table-2 . Considering only patients who filled out the questionnaire, 77.8% followed the recommended physiotherapy protocol using the vacuum at least two times per day, every day, until the second stage of surgery. Conversely, 22.2% patients used the vacuum less than two times per day or less than 7 days a week or had interrupted the vacuum therapy before the second stage of surgery. According to parent’s response, 93.3% felt that the use of the vacuum was easy or moderately easy. On other hand, 6.7% of the parents opined that the physiotherapy was challenging. According to the parent’s assessment, 26.7% children did not complain during the vacuum physiotherapy, 60.0% of children complained only at the beginning of the physiotherapy and 13.3% of children complained every time during the physiotherapy. None of the parents interrupted the physiotherapy because it was too difficult or because their son couldn’t stand the procedure. In response to the question, “Would you undergo the vacuum physiotherapy again?” 100% of the parents replied yes.

**Table 2:** Summary of the responses to our survey on the use of vacuum device between the two stages of BMG urethroplasty.

Questions	Answers			

Patients who replied to the questionnaire (45/59, 76.3%)				
**Did you use the vacuum device between the two stages of surgery?**	**Yes**	**No**		
Response rates	45 (100%)	0 (0%)		
**How many days per week did you use the vacuum device? (Days per week)**	**7-6**	**5-3**	**2-1**	
Response rates	44 (97.8%)	0	1 (2.2%)	
**How many times per day did you use the vacuum device?**	**3**	**2**	**1**	
Response rates	9 (20.0%)	30 (66.7%)	6 (13.3%)	
**For how long after the first surgery did you use the vacuum device? (Months)**	**6 (or until the second stage )**	**3-4**	**2-1**	**≤1**
Response rates	41 (91.1%)	1 (2.2)	2 (4.5%)	1 (2.2)
Number of patients, who followed our recommended protocol vacuum physiotherapy (at least two times per day, every day, until the second stage of surgery)				35 (77.8%)
**Did you have any difficulties in using the vacuum device?**	**No, it was easy**	**It was moderately easy**	**It was challenging**	**I interrupted because it was too difficult**
Response rates	32 (71.1%)	10 (22.2%)	3 (6.7%)	0 (0%)
**How was your son reacting to the vacuum device?**	**He never complained**	**He was complaining, but only at the beginning**	**He was complaining at all times**	**I interrupted because my son could not tolerate the vacuum**
Response rates	12 (26.7%)	27 (60.0%)	6 (13.3%)	0 (0%)
Number of parents who found vacuum physiotherapy was easy or moderately easy combined with children who did not complain or complain but just at the beginning				39 (86.7%)
**Did you perform the massage of the ventral side of the penis, over the buccal mucosa graft, with the cream?**	**Yes**	**No**		
Response rates	44 (97.8%)	1 (2.2%)		
**Would you perform physiotherapy with the vacuum device again?**	**Yes**	**No**		
Response rates	45 (100%)	0 (0%)		

Success rate of graft tubularization, rate of straight penis after two-stage urethroplasty and status of early (4 months) postoperative urinary stream

In 58/59 (98.3%) patients, BMGs were considered smooth and trophic or “favorable for tubularization” and these patients underwent second stage surgery. Only one graft was considered “unfavorable for tubularization”. For this patient, we harvested a new BMG and tubularized the plate in same sitting. This patient had not followed our protocol for the vacuum physiotherapy. The physiotherapy was performed less than two days per week, only one time per day and only for one month after the first stage of surgery. After a median follow-up of 18 months from the second stage of surgery, 52/59 (88.2%) patients presented a straight penis (<10° of PC) and none underwent surgery for recurrent PC. As part of the first stage of surgery, tunica transverse incisions were performed in all 59 patients, after urethral plate transection, because of PC. Preoperatively, 50 (84.7%) patients had severe (>60°) PC, 8 patients (13.6%) had moderate (30-60°) PC and only one (1.7%) had mild (<30°) PC. At second stage and after vacuum physiotherapy, 11 (18.6%) patients had no recurrent PC and did not receive any further correction. Conversely, 6 (10.2%), 38 (64.4%), and 4 (6.8%) had <30°, 30-60° and 60 degrees of recurrent PC and underwent dorsal plication. Overall, in 50/59(84.7%) patients the severity of recurrent PC at second stage was lower that at initial presentation. Additionally, of those 50 patients with severe (>60°) PC at presentation, 46 (92%) had less severe recurrent PC (<60°) at the second stage. Specifically, 34/50 (68%) had 30-60°, 4/50 (8%) had <30° and 8/50 (16%) had no residual PC. Last but not least,47/59 (79.7%) patients presented a powerful and straight urinary stream, four months after the second stage of surgery. Whereas, 12 (20%) patients had a weak or deflected urinary stream, as per the subjective assessment of two team members.

### Post-operative complications

After a median follow-up of 18 months from the second stage of surgery, 30 (50.8%) patients experienced one or multiple post-operative complications. The most frequent complication was postoperative urethro-cutaneous fistula (n=27, 45.8%), followed by urethral diverticulum (n=5, 8.5%) and urethral stricture (n=2, 3.4%).

## DISCUSSION

In this study we aimed to assess the feasibility and possible complications of vacuum physiotherapy protocol adopted by us. Three-fourth of all parents closely followed the recommended protocol of physiotherapy. Moreover, neither the parents nor the patients found the physiotherapy unpleasant, so as to justify the discontinuation. These results dispel the notion that the use of the vacuum device may be considered invasive and unpleasant for a two-year old child. On the other hand, the adherence rate to the protocol was encouraging, without interruptions due to parent or patient complain or complications associated with the procedure. Parent satisfaction was high in spite of the stringent physiotherapy protocol adopted (vacuum device was recommended twice a day, every day, for six months). Indeed, all parents replied that they would have repeated the physiotherapy, if they had to. These results prove beyond doubt that our protocol is practically feasible and safe for the parents and patients. No injury to the BMG or the operated genitals was seen during this study. Attention and time were devoted to provide the patients with proper instructions and useful tips.

The success rate of tubularization, at the time of second stage, was 98.3% (58/59 patients). The only unsuccessful patient was the one who did not follow our physiotherapy protocol. Though it is conceptually tempting to attribute this failure to lack of vacuum physiotherapy, our study was not designed to assess the possible causal association of vacuum physiotherapy with outcomes. Nonetheless, our results compared favorably with previous reports ( 4 , 14 - 16 ), where success rate of tubularization was between 94% and 87% of cases.

Noteworthy, PC at second stage was less severe than PC at presentation in 86.2% of patients. Such rate, was even higher in patients with initial severe PC (>60%), where 92% of these presented less severe recurrent PC after vacuum physiotherapy. Additionally, 18.6% of patients had no recurrent PC at all. These results are remarkable considering that ventral incisions are always associated with scar formation, contraction, and consequently recurrent PC, if grafts are not applied over the tunica incisions ( 17 - 19 ). In consequence, the vacuum physiotherapy was crucial in our patients to consolidate the result obtained after tunica incisions by limiting the scar formations, hence reducing the severity of recurrent PC, as well as the number of plications needed at second stage. Taken together, we can postulate that the vacuum physiotherapy has played an important role in achieving a straight penis. This has been corroborated by our final rate of absence or limited PC (<10°) in 88% (52/59) of our patients.

The early (4 months) post-operative urinary stream was powerful and straight in approximately 80% of patients. The good urethral plate for second stage should logically result in good lumen, pliant urinary conduit resulting in good urine flow post-operatively. However, we acknowledge that this outcome is dependent on multiple variables. The use of vacuum device resulting in good uroflow outcomes, is our speculation derived from our clinical experience, however, in absence of a control arm it is not possible to test it.

To assess the generalizability of our patient cohort, we have reported outcomes which were not part of study aim e.g. complications. It can be argued that the rate of post-operative complications after the second stage was considerably higher than in reported literature ( 20 ). Indeed, we reported a 50.8% rate of overall complications and, more specifically, 45.8% rate of urethro-cutaneous fistula. However, it should be taken into account that we included only patients with proximal hypospadias, and 13/59 (22.0%) patients had a history of previous failed hypospadias repair. In essence, these patients represented 20% (60/301) of most complex surgical procedures amongst all hypospadias surgeries that we did in last 5 years. Instead, we feel this complication rate though needs our attention, actually is result of near-complete follow-up and honest reporting. Furthermore, it would require some imagination to attribute the use of vacuum device to post-operative urinary fistulas after second stage repair. Instead, this intervention was designed to facilitate the tubularization avoiding re-grafting after the first stage. Finally, we did find some other investigators in this field having experience similar to ours. McNamara et al. reported a 50% rate of complications or reoperation in their cohort of 134 patients, who underwent two-stage hypospadias repair ( 21 ).

In our loss to follow-up analysis ( Table-1 ), we did not find any difference in known characteristics between the group of patients who responded and those which did not. Rather, the surgical outcomes (urinary fistula) were better in group which did not return the filled questionnaire. It is difficult to imagine the reason why the response of this group would have been different from the one which filled and returned the questionnaire.

Some limitations of the study need to be acknowledged, so as to interpret the study results objectively. First, the questionnaire did not undergo external validation. The evaluation of the relevance and completeness of our questionnaire is based on subjective judgment and could vary by researcher or setting perspective. Second, the number of patients included was relatively small, as it was for other study examining proximal hypospadias surgery ( 22 ). The feasibility of the vacuum physiotherapy protocol may vary when applied to larger patient group or different clinical set up. On the other hand, proximal hypospadias is a rare condition compared to distal hypospadias. Reaching an appropriate number of patients may require many years especially when they are enrolled in only one center, hence a multi-institutional study assessing the feasibility of protocol is the next logical step. Lastly, the design of the study was not meant to assess the association of the intervention with outcomes, as it did not include a control group. However, in light of facts that this non-invasive intervention is cost-effective and not associated with any harm, it is an appropriate candidate for further assessment, even if expected benefits are assumed to be modest. However, we felt it is unethical, to compare nothing vs. physiotherapy with vacuum, which has demonstrated its safety and benefits in other urological and pediatric conditions ( 23 ). For this reason, we preferred to run a cohort study, where all patients were counseled to receive the best medical support available, despite it being not yet recommended.

## CONCLUSIONS

Vacuum physiotherapy performed by parent of the patients is practically feasible, safe and easy to use. Our vacuum physiotherapy protocol had high compliance rate (77.8%). Vacuum physiotherapy is an appropriate candidate for further assessment in patients undergoing two stage hypospadias repair using buccal mucosa.

## ABBREVIATIONS

DSD: Disorders of sex development
TIP: Tubularized incised plate urethroplasty
IQR: Interquartile ranges
BMG: Buccal mucosa graft
PC: Penile Curvature
CI: Confidence Interval

## APPENDIX

Supplementary Video 1 - The current video shows how to use the vacuum device. The same video was used as example for all parents that were instructed on the physiotherapy.

https://intbrazjurol.com.br/videos/20190845_Bandini_et_al.mp4
